# Editorial: Resilience Resources in Chronic Pain Patients: The Path to Adaptation

**DOI:** 10.3389/fpsyg.2019.02848

**Published:** 2019-12-20

**Authors:** Carmen Ramírez-Maestre, Rocío de la Vega, John Andrew Sturgeon, Madelon Peters

**Affiliations:** ^1^Andalucía Tech, Facultad de Psicología, Instituto de Investigación Biomédica de Málaga, Universidad de Málaga, Málaga, Spain; ^2^Center for Child Health, Behavior and Development, Seattle Children's Research Institute, Seattle, WA, United States; ^3^Department of Anesthesiology and Pain Medicine, University of Washington School of Medicine, Seattle, WA, United States; ^4^Department of Clinical Psychological Science, Faculty of Psychology and Neuroscience, Maastricht University, Maastricht, Netherlands

**Keywords:** chronic pain, resilient resources, adaptation, wellbeing, positive psychology

Individual differences affect the behavior of individuals in response to chronic pain, and such behaviors can lead to either disability or capacity (Ramírez-Maestre and Esteve, 2013). Modern theories of adaptation necessitate a greater emphasis on individual differences in behavioral patterns oriented toward meaningful function despite pain, as well as effective recovery from the negative impacts of pain. Recent empirical studies have acknowledged the positive influence of resilience resources on adjustment to chronic pain (Sturgeon and Zautra, 2016; Hemington et al., 2017; Arewasikporn et al., 2018; Esteve et al., 2018; Ramírez-Maestre et al., 2019). According to these investigations, resilience has emerged as a personal resource that increases the patients' capacity to manage pain effectively. Thus, despite having chronic pain, a resilient individual will be able to experience positive emotions and maintain a higher level of functioning. Patients with these positive characteristics may use more effective coping strategies, have better goal-adjustment, demonstrate greater levels of flexibility and acceptance across situations, and maintain an appropriate functioning level. The studies collected in the present Research Topic are good examples of novel investigation in this context.

There are nine manuscripts in this special issue, including eight original research studies and one narrative review. Three papers analyze the role of positive variables in different stages of the lifespan: Childhood, adolescence, and older adulthood.  Hynes et al. review paper identifies family characteristics that are associated with both risk and resilience in children with Juvenile Idiopathic Arthritis (JIA). Their study, which includes seven articles in a narrative review, delineates the contribution of individual and parental resilience mechanisms and resources to resilience outcomes in children with JIA and their families. Hynes et al. highlight children's psychological flexibility, self-efficacy, treatment adherence, pain acceptance, and perceived social support as key contributors to resilience outcomes. The study of  Beeckman et al. examines the role of parental protective responses and instructions to engage in activities in adolescents' daily pain-related behavior. The results show an (indirect) adaptive role of parental psychological flexibility on adolescent daily pain-related behavior via its impact on parental protective behavior, highlighting the dynamic, and transactional nature of pain adaptation among adolescents in a family system. Finally,  Bartley et al. examined the association of multisystem resiliency with pain and psychological outcomes in a sample of older adults with chronic low back pain (cLBP). The aims were to empirically identify domains of resilience based upon psychological, social, and health-related factors and, using cluster analysis,explore whether resiliency phenotypes differ across measures of physical function, pain intensity, disability, and psychological functioning. Their findings indicate that individuals with a more resilient phenotype may present a greater sense of coherence that fosters successful mobilization of resources and navigation of ongoing challenges associated with pain. This study is among a small number to suggest the presence of resiliency profiles based upon psychological, social, and health-related functioning and, further, it suggests that health and psychosocial factors are differentially expressed across older adults with cLBP.

Two papers analyzed the role of resilient variables in the adaptation of women with fibromyalgia. Pastor-Mira et al. aimed (1) to develop a Spanish version of the Goal Pursuit Questionnaire (GPQ); and (2) to explore the relationships between goal preferences and health outcomes, testing the moderating role of affect and the mediating role of chronic pain activity patterns in two studies. In Study 1, they adapted the GPQ to a Spanish-speaking population of women with fibromyalgia. The culturally adapted Spanish version resulted in a shorter version with content changes reflected in the list of proposed activities, as a result of the field testing within the target population. The findings of study 2 revealed that a preference for pain avoidance goals are related to pain, disability, and fibromyalgia impact through activity patterns. Affect ratings showed direct and indirect associations with health outcomes, mainly by increasing task-contingent persistence. The authors conclude that interventions should promote adaptive activity patterns (i.e., task persistence) and reduce activity avoidance.

Also in a sample of women with fibromyalgia, Cejudo et al. examined the effects of a mindfulness-based intervention (MBI) on the improvement of subjective well-being, positive affect, trait emotional intelligence (TEI), mental health, and resilience. A design of repeated measures with a control group (CG) was used: before and after the application of the treatment and a 6 months follow-up. The study demonstrates that MBI may be an effective intervention tool to foster resilience and related constructs.

The remaining four studies examine flexibility, pain-specific resilience, and frustration tolerance as adaptation resources for chronic pain patients, and flourishing as a measure of well-being.

Gentili et al. examined the role of psychological flexibility (measured as avoidance, value obstruction, and value progress) in relation to symptoms (pain intensity and anxiety), and functioning (pain interference and depression) among adults with chronic pain applying for participation in a digital ACT-based self-help treatment. Their results show that psychological flexibility, as a resilience factor, significantly contributed to the prediction of pain interference and depression when adjusting for age, pain, and anxiety.

In a study in people living with HIV and chronic pain, Gonzalez et al. report that greater pain-specific resilience is significantly associated with lower levels of pain interference and pain catastrophizing, greater use of distraction and coping self-statements, and significantly greater heat pain tolerance. Authors suggest that pain-specific resilience may promote adaptation and positive coping in people living with HIV and chronic pain.

In a longitudinal study, Suso-Ribera et al. assessed a sample of individuals with chronic pain at two time points: 2 weeks before starting medical treatment at a pain clinic, and 6 months after. Authors found a reduction in pain intensity and an improvement in physical functioning. The results of the regression analyses show that a decrease in pain intensity is significantly associated with improvements in physical health and that this association is moderated by frustration tolerance.

Finally, Trompetter et al.'s paper explores, in two samples of individuals with chronic pain, the prevalence and sociodemographic, physical and psychological correlates of flourishing, and complement this exploration with a similar examination of risk for psychopathology. Compared to those without chronic pain, people with chronic pain were as likely to flourish, but more likely to be at risk for specific indices of psychopathology. Both flourishing and risk for depression were related foremost to psychological correlates. While engaged living was the most important correlate of flourishing, pain catastrophizing and psychological inflexibility were the most important correlates of being at risk for depression, suggesting potentially distinct underlying pathways for resilience and vulnerability in chronic pain. The authors suggest the Psychological Flexibility model to explain both poor and optimal functioning in the presence of chronic pain.

## Conclusions

The papers included in this Research Topic demonstrate the positive contribution of a diverse array of psychological characteristics in the adaptation and well-being of individuals with chronic pain across the lifespan. Several clinical implications have been derived from the findings. More research is needed to further illuminate trajectories of effective pain management, which may have significant value for future clinical and empirical models in chronic pain.

## Author Contributions

All authors listed have made a substantial, direct and intellectual contribution to the work, and approved it for publication.

### Conflict of Interest

The authors declare that the research was conducted in the absence of any commercial or financial relationships that could be construed as a potential conflict of interest.
